# Evaluation of an Anti-Thrombotic Continuous Lactate and Blood Pressure Monitoring Catheter in an In Vivo Piglet Model undergoing Open-Heart Surgery with Cardiopulmonary Bypass

**DOI:** 10.3390/chemosensors8030056

**Published:** 2020-07-17

**Authors:** Kenneth Kwun Yin Ho, Yun-Wen Peng, Minyi Ye, Lise Tchouta, Bailey Schneider, McKenzie Hayes, John Toomasian, Marie Cornell, Alvaro Rojas-Pena, John Charpie, Hao Chen

**Affiliations:** 1 Biocrede Inc., Plymouth, MI 48170, USA;; 2 Division of Pediatric Cardiology, Department of Pediatrics, University of Michigan, Ann Arbor, MI 48109, USA;; 3 Extracorporeal Life Support Laboratory, Department of Surgery, University of Michigan, Ann Arbor, MI 48109, USA;; 4 Section of Transplantation, Department of Surgery, University of Michigan, Ann Arbor, MI 48109, USA

**Keywords:** blood lactate, continuous monitoring, lactate sensors, congenital heart disease, cardiopulmonary bypass, cardioplegic ischemia, cardioplegic reperfusion, intravenous

## Abstract

Blood lactate and blood pressure measurements are important predictors of life-threatening complications after infant open-heart surgeries requiring cardiopulmonary bypass (CPB). We have developed an intravascular nitric oxide (NO)-releasing 5-Fr catheter that contains a lactate sensor for continuous in-blood lactate monitoring and a dedicated lumen for third-party pressure sensor attachment. This device has antimicrobial and antithrombotic properties and can be implanted intravascularly. The importance of this design is its ability to inhibit thrombosis, due to the slow release of NO through the surface of the catheter and around the electrochemical lactate sensors, to allow continuous data acquisition for more than 48 h. An in vivo study was performed using six piglets undergoing open-heart surgery with CPB and cardioplegic arrest, in order to mimic intra-operative conditions for infants undergoing cardiac surgery with CPB. In each study of 3 h, two 5-Fr NO-releasing lactate and blood-pressure monitoring catheters were implanted in the femoral vessels (arteries and veins) and the CPB circuitry to monitor changing lactate levels and blood pressures during and immediately after aortic cross-clamp removal and separation from CBP. Electrical signals continuously acquired through the sensors were processed and displayed on the device’s display and via Bluetooth to a computer in real-time with the use of a two-point in vivo calibration against blood gas results. The study results show that lactate levels measured from those sensors implanted in the CPB circuit during CPB were comparable to those acquired by arterial blood gas measurements, whereas lactate levels measured from sensors implanted in the femoral artery were closely correlated with those acquired intermittently by blood gas prior to CPB initiation, but not during CPB. Blood pressure sensors attached to one lumen of the device displayed accurate blood pressure readings compared to those measured using an FDA approved pressure sensor already on the market. We recommend that the sensor be implanted in the CPB’s circuit to continuously monitor lactate during CPB, and implanted in the femoral arteries or jugular veins to monitor lactate before and after CPB. Blood pressures dramatically drop during CPB due to lower blood flow into the lower body, and we suspect that the femoral arteries are likely collapsing or constricting on the implanted catheter and disrupting the sensor-to-blood contact. This study shows that the device is able to accurately and continuously monitor lactate levels during CPB and potentially prevent post-surgery complications in infants.

## 1. Background

The “holy grail” in the field of biomedical sensors is the development of implantable intravascular in-blood sensors used in the real-time monitoring of important physiological chemical species in humans. Devices that can accurately measure blood pH, carbon dioxide, oxygen, electrolytes (Na^+^, K^+^, and Ca^2+^), glucose, and particularly lactate levels on a continuous basis could dramatically change the way in which critically ill patients are managed in intensive care units (ICU) [1–3]. Hyperlactatemia, defined as lactate levels of >1.5 mmol/L in patients, has been shown to be a predictor of high mortality in a variety of critically ill patients, including infants after open-heart surgery, and adults with sepsis, cardiac arrest, trauma, organ failure, or acute respiratory distress syndrome [4–16]. Furthermore, lactate levels are being used to monitor the success of early goal-directed therapies for the resuscitation of sepsis patients with elevated lactates [17–19]. There is increasing evidence suggesting that the normalization of traditional hemodynamic parameters is sometimes unreliable and inappropriate for individual patients with a different physiology [20–22], while blood lactate levels seem to be more relevant to the resuscitation outcome in the early phase [23–26]. Findings confirm that lactate monitoring is a valuable parameter in the early resuscitation of critically ill patients with organ failure and sepsis [4,27–30], and lactate-assisted therapy significantly reduced hospital mortality in patients with hyperlactatemia on ICU admission when lactate levels were decreased by >20% [4,28].

Despite extensive research and development efforts for many years [31–40], there remain significant technical challenges in continuous lactate measurements, and, thus, there are currently no intravascular chemical sensors commercially available for the continuous real-time monitoring of blood lactate in ICU patients. During surgery, an assessment of blood lactate is typically performed with a blood gas analyzer (ABL800 Flex blood gas analyzer, Radiometer America, Brea, CA) using discrete blood samples drawn from existing intravenous (IV) or intra-arterial lines. Therefore, many research groups have been developing continuous in vivo electrochemical sensors for parameters such as glucose and lactate since the early 1990s/2000s [33,35,37–39,41–45]; however, biocompatibility and coagulation challenges lead to significant accuracy problems [31,32,34,46,47]. A common observation for sensors (electrochemical or optical) during in vivo studies is a performance pattern in which, after a given period of time, sensor output signals often significantly deviate from the values obtained for discrete blood samples analyzed in vitro. The pattern is always the same, with IV sensor values for PO_2_ (and pH) always being lower than corresponding in vitro measurements. Similar errors are observed for glucose measurements [48]. This can be attributed to the adhesion of activated platelets that form a blood clot on the surface of implanted sensors, in which platelets and other entrapped cells create a local environment that differs in PO_2_ and lactate/glucose levels compared to the bulk blood (due to cellular respiration and the consumption of oxygen/glucose). Therefore, no continuous real-time intravenous lactate sensors for ICU or surgical patients are commercially available yet. The use of IV catheters alone (w/o sensors) is a major source of infection in the hospital setting, with a staggering 28,000 deaths associated with catheter infections annually in the US [49,50]. Biofilm formation is often the cause of catheter-associated blood stream infections.

Congenital heart disease (CHD) is the most common type of birth defect, occurring in nearly 1 per 110 live births in the United States, and more than 1.3 million neonates annually in the world. Nearly 25% of these infants will require a surgical intervention within the first two years of life [3], and approximately 8.2% of these surgical procedures will be associated with adverse outcomes, such as extracorporeal membrane oxygenation (ECMO) (3.9%), acute kidney injury requiring dialysis (3.0%), and death (4.8%) [1,2]. Despite recent innovations in cardiopulmonary bypass (CPB) and myocardial preservation, postoperative myocardial dysfunction and end-organ injury remain leading causes of morbidity and mortality. While it is still unclear why myocardial dysfunction occurs following open-heart surgery, blood lactate levels during and after CPB are predictors of major complications after cardiac surgeries and are employed to identify patients with higher risks of morbidity and mortality [1,2,51,52]. If blood lactate levels were continuously monitored and an abnormal lactate rate increase was quickly detected, first-line treatments such as packed red blood cell (PRBC) transfusion to increase hemoglobin and the O_2_ carrying capacity, systemic cooling, sedation, and neuromuscular blockade to decrease oxygen consumption, as well as the institution of vasoactive-inotropic drugs such as milrinone to increase contractility and decrease systemic vascular resistance, could be implemented earlier to more effectively prevent major morbidity and mortality. Findings have confirmed that lactate monitoring is a valuable parameter in the early resuscitation of critically ill patients with organ failure and sepsis, and lactate-assisted therapy significantly reduced hospital mortality in patients with hyperlactatemia on ICU admission when lactate were decreased by >20% [4,29]. Furthermore, postoperative glucose monitoring in the early intervention of hyperglycemia reduces surgical site infection and hyperglycemia-related complications [53–57].

We have developed a wire-type, immobilized enzyme-based electrochemical lactate sensor mounted within a polymeric, low-profile nitric oxide (NO)-releasing catheter tethered to a circuit module to wirelessly monitor lactate levels and store data [58]. The catheter-housed sensor has antithrombotic properties through the application of S-nitroso-N-acetylpenicillamine (SNAP) doped in the polymeric catheter. When the catheter-housed sensor is exposed to moisture and body temperature, NO is released from the catheter surfaces, resulting in antimicrobial [59,60] and antithrombotic properties to prevent infection and thrombosis/fibrin sheath formation on the sensor [46,47,61–65]. We have evaluated the continuous blood lactate measurements of the catheter-housed sensor within the jugular and femoral veins of heparinized [58] and non-heparinized (unpublished) porcine models under anesthesia, and showed effective continuous lactate measurements every minute over 10 h. To better mimic postoperative cardiomyocyte injury, ventricular dysfunction, and metabolic derangements with elevated lactates in neonates following open-heart surgery with CPB, we performed in vivo intravenous implantations of catheter-housed lactate sensors in piglets undergoing CPB, followed by induced cardioplegic ischemia and cardioplegic reperfusion.

## 2. Materials and Methods

### 2.1. Materials

Lactate oxidase (from *Aerococcus viridans*), sodium L-lactate (sodium salt), glutaraldehyde, bovine serum albumin (BSA), fetal bovine serum (filtered and sterilized), phosphate buffered saline, iron (III) chloride (FeCl_3_), 37% hydrochloric acid (HCl), Nafion (5 wt% solution in lower aliphatic alcohols/H_2_O solution), 1,3-diaminobenzene, resorcinol, poly(ethyleneimine) solution (PEI), glycerol, tetrahydrofuran (THF), and Pluronic F-127 were all obtained from Sigma-Aldrich (St. Louis, MO, USA). Perfluoroalkoxy alkane (PFA)-coated platinum/iridium and silver wires were obtained from A-M Systems (Sequim, WA, USA). E2As Elast-Eon polyurethane was a gift from Aortech Biomaterials (Weybridge, Surrey, UK). 5-Fr double lumen central venous catheterization kits were obtained from Sungwon Medical (Cheongju-si, Korea).

### 2.2. Sensor Fabrication and Catheter Assembly

The sensor relies on the chemical reaction of lactate oxidase with lactate to form hydrogen peroxide, and a platinum electrode reacts with the hydrogen peroxide to produce electrons. This generated current that is proportional to the concentration of lactate is then sensed by the processor within the potentiostat.

The fabrication of lactate sensors was improved from our previous study [58] and is based on previous designs [32,41,48,66,67]. Briefly, the sensor is comprised of two electrode wires, where the platinum/iridium wire serves as the working electrode and the silver/silver chloride wire serves as the reference electrode. A 1 mm length sensing region in the outer Teflon coating of the platinum/iridium wire was cut (outer diameter = 0.2 mm) to function as the working electrode area. A thin film Nafion coating was applied to the working electrode surface, and a layer of polymerized resorcinol and 1,3-diaminobenzene was then applied to the cavity through a cyclic voltage (CV) electropolymerization process (0 and +0.83 V at 0.002 V s^−1^ for 18 h) [68,69]. The polymerized layer promotes the rejection of electroactive interference species, such as ascorbic acid, uric acid, and acetaminophen, from reaching the working electrode region of the sensor surface, which can cause interfering background current. A silver/silver chloride (Ag/AgCl) electrode composed of a Teflon-coated silver wire treated with acidified ferric chloride solution was tightly coiled around the platinum/iridium wire in close proximity to the working electrode sensing region to serve as the lactate sensor’s electrochemical reference. Heat-shrinkable polyester tubing was applied to secure the reference wire in place and mechanically reinforce the sensor element assembly. Lactate oxidase enzyme, stabilized within a PEI solution, was immobilized in the cavity of the working electrode using glutaraldehyde. After the enzyme/glutaraldehyde crosslinking had formed, sensor outer layers consisting of a 5% (wt/vol) polyurethane and silicone rubber RTV (Silco Sil-Bond RTV 4500) in THF solution were coated to restrict analyte diffusion to the enzyme layer. The lactate sensors were tested against interference species and different concentrations of sodium L-lactate solutions to verify their linear functionality.

**Figure1.**
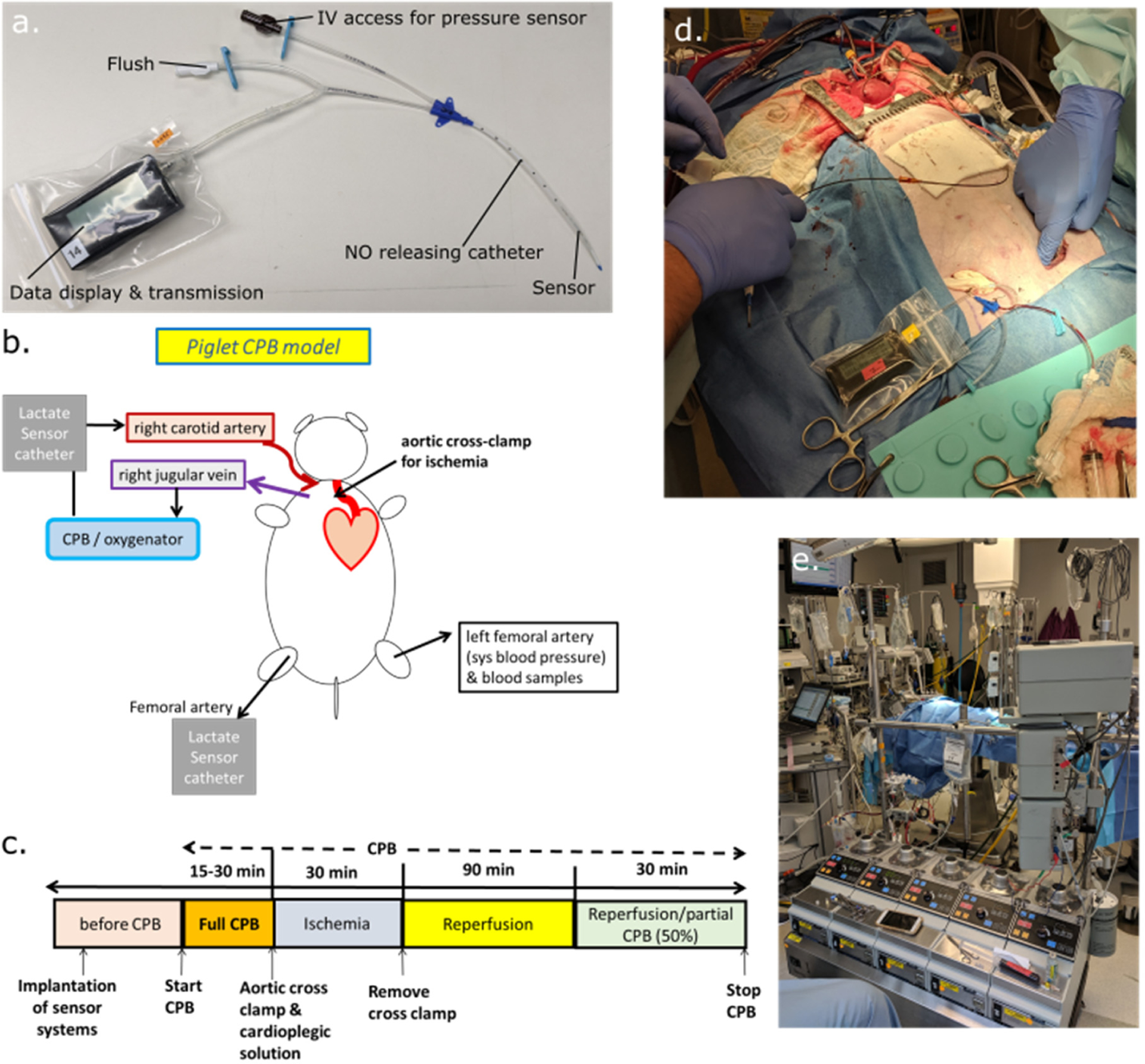
(**a**) An anti-thrombotic 5-Fr dual lumen central venous catheter with an integrated lactate sensor and Bluetooth-enabled potentiostat with display was implanted in the animal for 3 h. (**b**) Schematic of the experimental setup and cardiopulmonary bypass (CPB) circuit. (**c**) Schematic showing the protocol of the open-heart surgery with CPB. (**d**) Intravenous implantation of the device in the femoral artery of a piglet in vivo. (**e**) Clinical pediatric CPB circuit used in the study.

The lactate sensors were assembled inside a modified 5-Fr double lumen central venous catheter with SNAP suspended within the silicone-based polymeric material. In the presence of water and at body temperature, SNAP releases NO from the silicone and converts into a disulfide byproduct which stays within the silicone. One dedicated lumen was used for intravenous access and measurement of the blood pressure via an attached third-party pressure sensor. The 5-Fr double lumen central venous catheter was manufactured and assembled with Bluetooth-integrated potentiostat devices, electrodes made in-house, device housings, and operational device firmware/hardware at Biocrede Inc. The potentiostat acquires lactate electrical signals every millisecond and displays averaged values once every 6 s, and data is averaged every minute, stored, and displayed. Finally, the catheter-housed lactate sensors were UV-sterilized before they were used in the animal study. A picture of the catheter-housed lactate sensor and Bluetooth-integrated potentiostat device is shown in Figure 1a.

### 2.3. Animal Preparation for Cardiopulmonary Bypass, Induced Cardioplegic Ischemia, and Reperfusion

Animal study protocols were approved by the University of Michigan’s Committee on the Use and Care of Animals, and animals received humane care in accordance with the NIH Guide for the Care and Use of Laboratory Animals. The experimental setup, CPB circuit, and implantation location of our NO-releasing lactate sensor catheters are summarized in Figure 1b, while the protocol of the open-heart surgery with CPB is shown in Figure 1c. Six 1–2-month male or female piglets (8.5 ± 2.5 kg, Regular Domestic Swine) were used for this in vivo study and all experiments were conducted following the surgical procedures described below. All animals underwent 12 h of fasting with ad libitum access to water prior to surgery to reduce complications during the induction of anesthesia. The animals were moved to the surgical rooms using ULAM transport cages following ULAM transport guidelines and anesthetized by chemical or gas inhalation procedures. General anesthesia was induced with 5–7 mg/kg of Telazol (Tiletamine-zolazepam, Zoetis Inc., Kalamazoo, MI, USA) combined with 2–3 mg/kg of Xylazine (Akorn, Inc., Lake Forest, IL, USA) given intramuscularly. Animals were intubated with a 4–5 mm ID cuffed endotracheal tube (Telefelx Medical, Inc., Research Triangle Park, NC, USA) and mechanical ventilation was instituted using a ventilator (Datex_ohmeda, AS5 Aestiva) with the fraction of inspired O_2_ (FiO_2_) at 50–70%, tidal volume set at 7–8 mL/kg, respiratory rate of 12–16 breaths/min, and peak end-expiratory pressure of 5–8 cmH_2_O. The minute volume was adjusted to maintain the PaCO2 at 40 ± 5 mmHg and peak inspiratory pressures at 19 ± 3 cmH_2_O. Anesthesia was maintained with a constant infusion of 1–3% inhaled anesthetic isoflurane (Piramal Enterprises ltd., Kohir Mandal, Andhra Pradesh, India).

Cutdowns were performed on the left femoral artery for intravenous access and measurement of the mean arterial pressure (MAP). Our 5-Fr central venous catheter with integrated lactate and pressure sensors was implanted in the right femoral artery and started monitoring blood lactate and MAP values (Figure 1d).

Sternotomy: Lidocaine (APP Pharma LLC, Schaumburg, IL, USA) was administered (0.5 mg/kg) before the skin incision. The pericardium was incised and tented with 0-silk traction stitches placed in the pericardium and skin for optimal exposure of the mediastinum and the great vessels. Purse-string sutures were placed in the aortic root and the right atrial appendage. A dose of 300 U/kg of unfractionated porcine heparin (Sagent Pharma, Schaumburg, IL, USA) was administered to increase the target activated clotting time (ACT) > 500 s. Following the administration of heparin, an 18-Fr single-stage venous cannula (Medtronic, Grand Rapids, MI, USA) was inserted through the right atrial appendage to provide venous drainage to the heart-lung machine. A 12-Fr pediatric one-piece arterial cannula (Medtronic, Grand Rapids, MI, USA) was inserted via an aortic purse-string into the ascending aorta to provide a return conduit from the CPB machine. For cardioplegia delivery, a purse-string was placed in the aortic root to insert a 4-Fr DLP aortic root cannula (Medtronic, Grand Rapids, MI, USA). The CPB circuit used was a basic clinical pediatric CPB circuit (Figure 1e) and consisted of a venous reservoir, roller pump, membrane oxygenator, and tubing for venous (drainage) and arterial (infusion) lines (FX05 Baby Capiox Oxygenator and reservoir- Terumo CVS, Ann Arbor, MI, USA). A CPB blood flow probe was attached just proximal to the reinfusion cannula to measure systemic blood flow. Two NO 5-Fr central venous catheters with integrated lactate sensors were placed in the CPB circuit proximal to the flow probe to monitor blood lactate values. Animals were supported intra-operatively during the procedure. Finally, a 5-Fr Millar pressure catheter was inserted transapical in the left ventricle (LV) for the continuous monitoring of LV pressures.

The global cardiac ischemic procedure was followed and CPB initiated at a full flow rate of 100 mL/kg/min. After 15 min of hemodynamic stability on CPB, a cross clamp was applied to the ascending aorta and 50 mL/kg of animal of high potassium Del Nido cold cardioplegia (CAPS Inc., Detroit, MI, USA) was infused antegrade via a cardioplegica cannula into the aortic root. After 30 min of cardiac ischemia, the aortic cross clamp was released and the heart was reperfused for 120 min. At the end of the studies of approximately 3–4 h, the animals were euthanized. Hemodynamic parameters, including the mean arterial pressure (MAP), heart rate (HR), left ventricular (LV) function, ECG, and rectal temperature, were monitored continuously using a computer equipped with a data acquisition system (PowerLab and LabChart, ADInstruments, Colorado Springs, CO, USA).

For electrolyte management, such as calcium gluconate, lactate, insulin, glucose, magnesium sulfate, potassium chloride, sodium bicarbonate, and other related drugs were administered by IV at any time within the study to bring blood values within normal ranges. During anesthesia and surgery, IV fluids were given to support the hemodynamics of the animal and replace any blood volume as needed. Blood gases were drawn throughout the duration of the experiment as needed by the surgeon before and after each major surgery step, sometimes random, or at least once every 30 min, to help monitor the animal pH, electrolyte, metabolite balance, etc. We used the first two animal tests to analyze our NO-releasing catheter sensor design to improve its blood pressure acquisition performance and stability.

## 3. Results

### 3.1. Lactate Measurement Results

**Figure 2.**
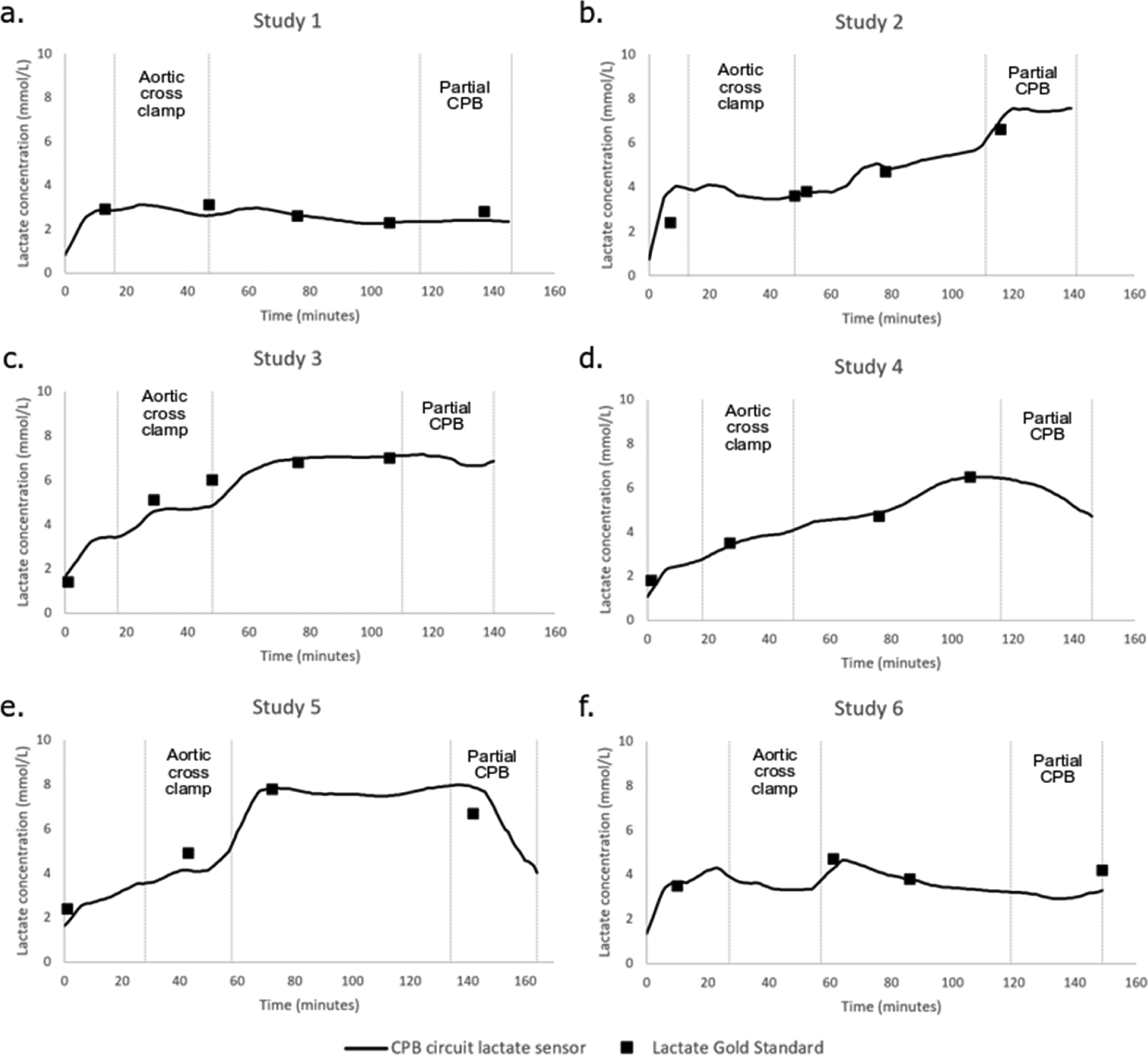
Continuous time trace of continuous lactate sensor measurements conducted in the cardiopulmonary bypass (CPB) circuits of the piglets in vivo (solid lines) compared to the control measured by blood gas (square markers), for each study (**a**–**f**), respectively. CPB initiated at time = 0.Aortic cross clamping was applied for 30 min to induce cardioplegic ischemia and released to allow reperfusion, and partial CPB was initiated in the last 30 min of reperfusion to wean off CPB support. In this figure, 2.5 h of each of the 3–4 h lactate studies are shown with desired lactate level changes, the results of which were obtained using NO lactate sensors designed for in-blood implantations of 48 h.

The lactate sensors are housed within the lumen towards the distal end of the 5-Fr catheter and connected to a CPU-integrated potentiostat with memory and integrated Bluetooth to record lactate levels (nA) every minute. In this study, recorded sensor output values were subsequently converted to mmol/L using a two-point conversion factor based on discrete lactate concentration values provided by a blood gas analyzer (ABL800 Flex, Radiometer America, Brea, CA, USA). Time traces of continuous lactate sensor measurements within the CPB circuits of the six piglet in vivo models are shown in Figure 2 for each study. Blood lactate levels of the piglets were allowed to equilibrate before CPB initiation (time = 0), leading to gradual increases of blood lactate concentrations via blood gas measurements and shown as solid squares in Figure 2. Lactate levels were null in the CPB circuit before CPB initiation due to non-blood recirculation. Continuous lactate sensor measurements in the CPB circuit correlated with those measured with the blood gas machine during the entire CPB, including cardioplegic ischemia and reperfusion via the application and release of the aortic cross clamp, respectively. Lactate levels measured by sensors implanted in the femoral arteries of the piglet in vivo models correlated with blood gas measurements prior to CPB initiation, previously shown in a study [58], but did not correlate during CPB. We recommend that the sensor be implanted in the CPB circuit to continuously monitor lactate during CPB, and implanted in the femoral arteries or jugular veins to monitor lactate before and after CPB. Since blood pressures measured at the femoral arteries dramatically drop during CPB due to lower blood flow, we suspect that the vessels slightly collapsed on the implanted catheter, disrupting sensor-to-blood contact.

### 3.2. Continuous Monitoring of the Blood Pressure

**Figure 3.**
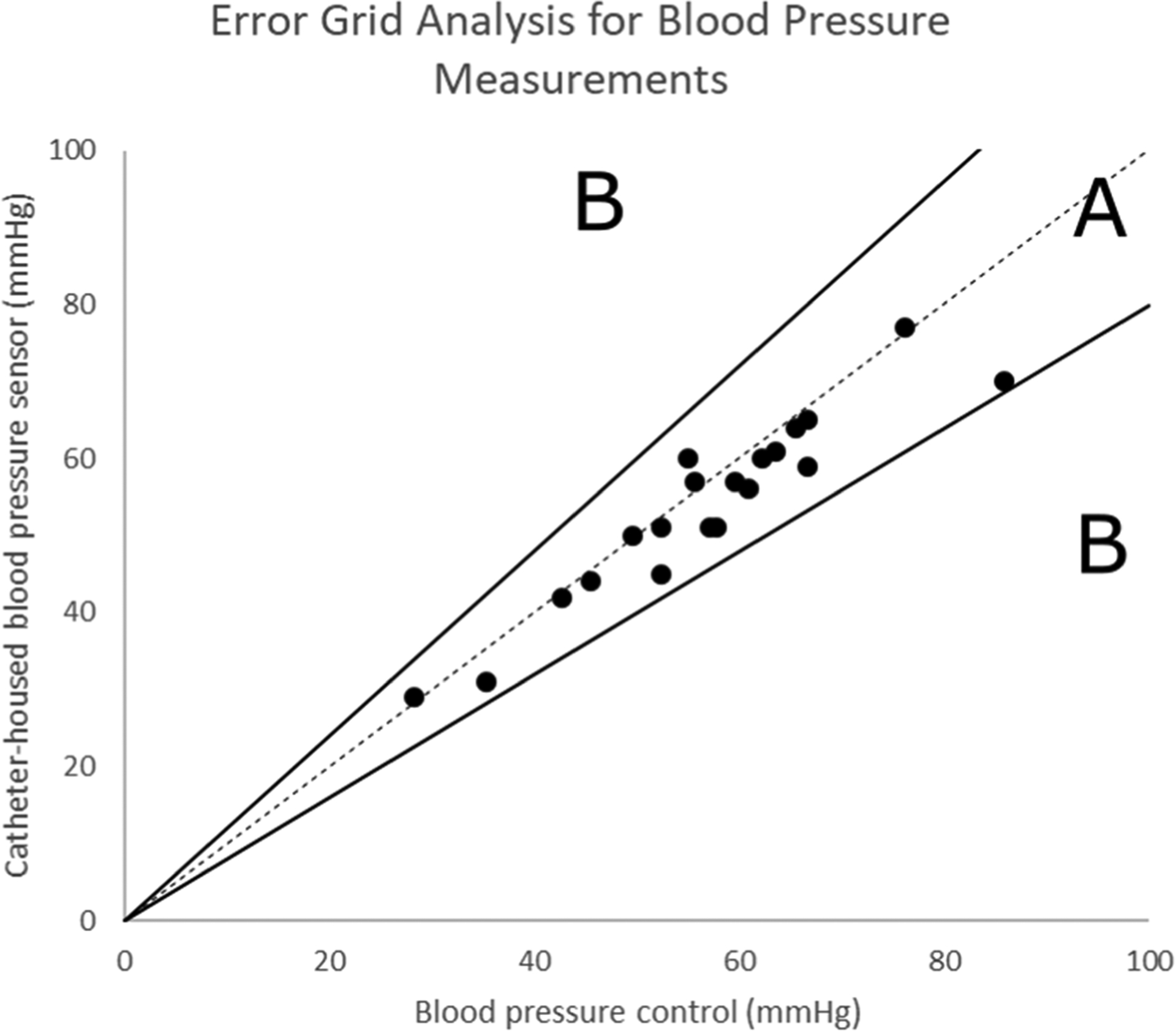
Error grid analysis of the blood pressure measured from the NO-releasing catheter with integrated lactate sensors implanted in the femoral arteries, compared to the control measured though an FDA-approved cannula implanted in another femoral artery. Zone A denotes accurate pressure measurements falling within 20% of error compared to the control. We used Clarke’s error grid analysis, which is typically used to quantify the clinical accuracy of blood sensors [70].

Each 5-Fr dual lumen CVC implanted in the femoral arteries had a dedicated intravenous access line connected to a blood pressure sensor to display the real-time pressure during open-heart surgery. An additional FDA-approved intravenous access line was implanted in a separate femoral artery to monitor the blood pressure as the control. The blood pressure sensor displayed the blood pressure in real-time on a monitor screen, but blood pressure measurements were not continuously annotated. These non-embedded third-party FDA-approved pressure sensors were attached to the designated lumen of our device. Discrete measurements were acquired by comparing blood pressure data acquired through our device and the control, and Clarke’s error grid analysis was performed. This analysis is typically used to quantify the clinical accuracy of blood sensors, such as those of glucose sensors [70]; the author of this study justifies why blood parameter estimates within 20% compared to reference values are clinically accurate (Figure 3). Blood pressure sensors attached to our NO-releasing sensor catheters showed a high accuracy amounting to 100% falling within the accepted Zone A and none in Zone B.

## 4. Discussion

### 4.1. Accuracy and Performance of Lactate Sensors

**Figure 4.**
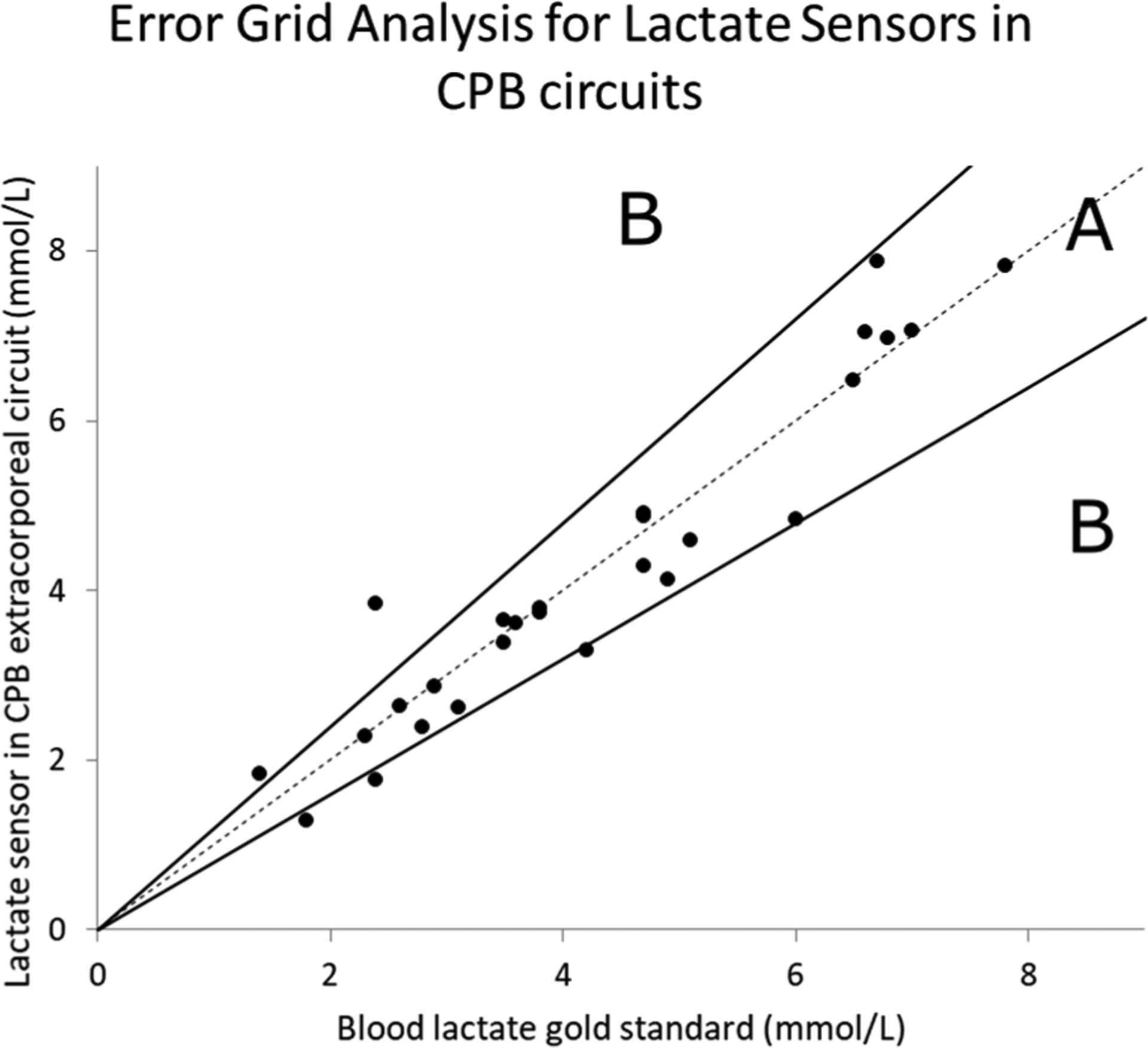
Error grid analysis of lactate measured with NO-releasing lactate sensors compared to that measured using a blood gas analyzer as the control. Zone A depicts a 20% proportional deviation between study and control measurements. The data point (2.4, 3.84) belonged to the first discrete blood gas comparison point in Study 2 (Figure 2b) and is considered an outlier. We used Clarke’s error grid analysis, which is typically used to quantify the clinical accuracy of blood sensors [70].

In a prior study, Biocrede Inc. showed that the implantation of 5-Fr catheter-housed lactate sensors in the femoral and jugular veins in porcine resulted in an acceptable clinical accuracy [58].To further demonstrate that lactate sensors can be implanted in CPB circuits to continuously monitor blood lactate levels during CPB, the results were compared to those of the control obtained from discrete blood gas measurements from the CPB circuit to evaluate the accuracy and performance. Lactate values from sensors implanted in the CPB circuits showed gradual increases in blood lactate during CPB, cold cardioplegic ischemia, and reperfusion and corroborated with the control. These sensors have a mean absolute percentage error of 11.1% ± 2.6%, which is better than other values found in the literature [48]. Clarke’s error grid analysis method [70] was used to build an error grid analysis on the sensors’ blood lactate measurements (Figure 4) and showed an acceptable accuracy for all biosensors falling within the acceptable range of Zone A. The data point (2.4, 3.84) belonged to the first discrete blood gas comparison point in Study 2 (Figure 2b); this discrete blood gas measurement did not seem to have measured the lactate overshoot steadily measured by the continuous sensor that the second blood gas measurement showed. We suspect that our team recorded the first blood gas blood-withdrawal time in Study 2 erroneously (human error).

### 4.2. Lower Body Hypoperfusion during CPB Interferes with Lactate Sensing

Previous studies conducted at Biocrede Inc. using NO lactate sensors showed that those implanted in pigs’ jugular veins showed faster lactate measurements compared to those implanted in the lower body’s femoral veins [58], with an average lag of 8–9 min. In this study, apart from implanting these NO-releasing lactate sensors in the CPB circuits, another set was implanted in piglets’ femoral arteries due to the limited real estate left in the jugular veins required to perform central CPB cannulation. The results show that the lactate levels measured in the femoral arteries correlated with blood gas measurements prior to CPB initiation, which is comparable to our previous study [58], but did not sense the gradual increases of lactate after CPB initiation.

While sensors in the CPB circuitry showed an acceptable accuracy compared to those measured by the blood gas analyzer, lactate values measured in the femoral artery sensors did not correlate with blood gas measurements during CPB. The blood pressure monitored through a pressure sensor in an intravenous access line implanted in a separate femoral artery using a computer data acquisition system (PowerLab and LabChart, ADInstruments, Colorado Springs, CO, USA) showed a decrease from an average of 58.5 ± 3.5 mmHg to an average of 34.0 ± 2.3 mmHg, 15 min before and after CPB initiation respectively. Based on these observations, our animal surgeon noted that the piglets displayed hypoperfusion in the lower parts of their bodies during CPB. Furthermore, intravenous access lines were inserted into the aortas during cardioplegic ischemia and reperfusion after CPB to analyze the animals’ arterial blood pressures, and these showed higher pressures compared to those measured at the lower body’s femoral arteries in real time. Hence, we suspect that the significant lactate discrepancy measured between the CPB circuit and the femoral artery during CPB is due to lower body hypoperfusion leading to a possible collapse of the blood vessel walls that blocked the sensing window site. All experiments showed consistent lactate results before and during CPB, and all piglets had hypoperfusion in the lower parts of their bodies during CPB. Since the diameter of 5-Fr catheters is similar to that of piglets’ femoral arteries’, our animal surgeon advocated for a possible partial artery collapse onto the sensor cavity due to the lower blood pressure that prevented flowing blood from fully contacting the sensors.

### 4.3. Recommendations for Future Uses and Potential Risks

The CPB surgery proposed is similar to surgeries performed in neonates with CHD [2,71] and the positive results suggest that our device seems to be ready for the first round of human testing. Open-heart surgeries with CPB in piglets are not well-understood, and the results differ, irrespective of the use of these NO-releasing lactate catheters. Importantly, the implantable section of the catheter does not seem differ from typical 5-Fr catheters and thus poses little to no risk to the body. The allocated sensors are flush with the catheter’s outer surface, and directly touch the blood vessel tissue during and after implantation. Furthermore, ISO-based GLP biocompatibility studies were performed by Wuxi AppTec Inc. (St. Paul, MN, USA) and NAMSA (Northwood, OH) on NO-releasing catheters to assess their toxicity and irritation. The results show that these NO-releasing catheters exhibited the safest scores possible—zero (0) for the in vitro toxicity testing (0–2 = safe; 3–4 = toxic) on L-929 Mouse Fibroblast Cells and for in vivo testing in mice—and the lowest irritant scores possible (0) for the ISO irritation study in rabbits. The sensors were designed to release NO at the same or a slightly lower rate as that released by our body cells for 2–4 days. NO molecules help reduce thrombosis and inflammation at the implanted sites, and thus pose no risk to and display no invasiveness for the patient’s body.

## 5. Conclusions

Blood lactate measurements during open-heart surgeries with CPB are predictors of major post-operative complications in patients with a high risk of morbidity and mortality. Biocrede has developed an NO-releasing 5-Fr CVC with an embedded lactate sensor for in-blood implantations of 48 h due to the catheter’s NO anti-thrombotic properties [58]. In this study, we analyzed the continuous lactate levels present in the circuit during CPB in six piglets in vivo, in order to mimic typical postoperative cardiomyocyte injury that often occurs in neonates during open-heart surgeries. The results show that our sensors implanted in the CPB circuit resulted in effective continuous lactate (blood gas control) and blood pressure measurements during CPB with cardioplegic arrest. These NO-releasing lactate sensors had a mean absolute percentage error of 11.1% when implanted, and were qualified as clinically accurate. Furthermore, these dual lumen sensors accurately measured the blood pressure within 20% of discrepancy compared to an FDA-approved pressure sensor in a separate cannulated site, and thus also qualified as clinically accurate. However, when CPB was initiated in animals, significant hypoperfusion occurred in their lower bodies, and arterial low flow and low blood pressure (<40 mmHg) led to the possible caving of blood vessels, blocking the sensor’s acquisition site. Both lactate sensors and lumens for pressure sensors were durable and clinically accurate at detecting endogenous lactic acid production and the blood pressure during open-heart surgery using CPB. We recommend that the implantation of continuous lactate sensor catheters in femoral arteries be performed for post operation lactate monitoring, and implanted in the CPB circuit during CPB due to lower body hypoperfusion. In this way, hyperlactatemia can be quickly detected and first-line treatment can be adequately performed to prevent postoperative complications and reduce morbidity and mortality. This study provides a possible development of a continuous blood lactate sensor with a pressure lumen for commercial use in neonates with CHD recovering from the operation.
